# Supplementary material for: Consistent tumorigenesis with self-assembled hydrogels enables high-powered murine cancer studies
Source: Commun Biol. 2021 Aug 19;4:985. doi: 10.1038/s42003-021-02500-8 (PMC8376877; doi:10.1038/s42003-021-02500-8)
Supplement: Supplementary file 2 — Supplementary Information [file 42003_2021_2500_MOESM2_ESM.pdf]

Supplementary Figures: Consistent Tumorigenesis with Self-Assembled Hydrogels Enables High-powered Murine Cancer Studies

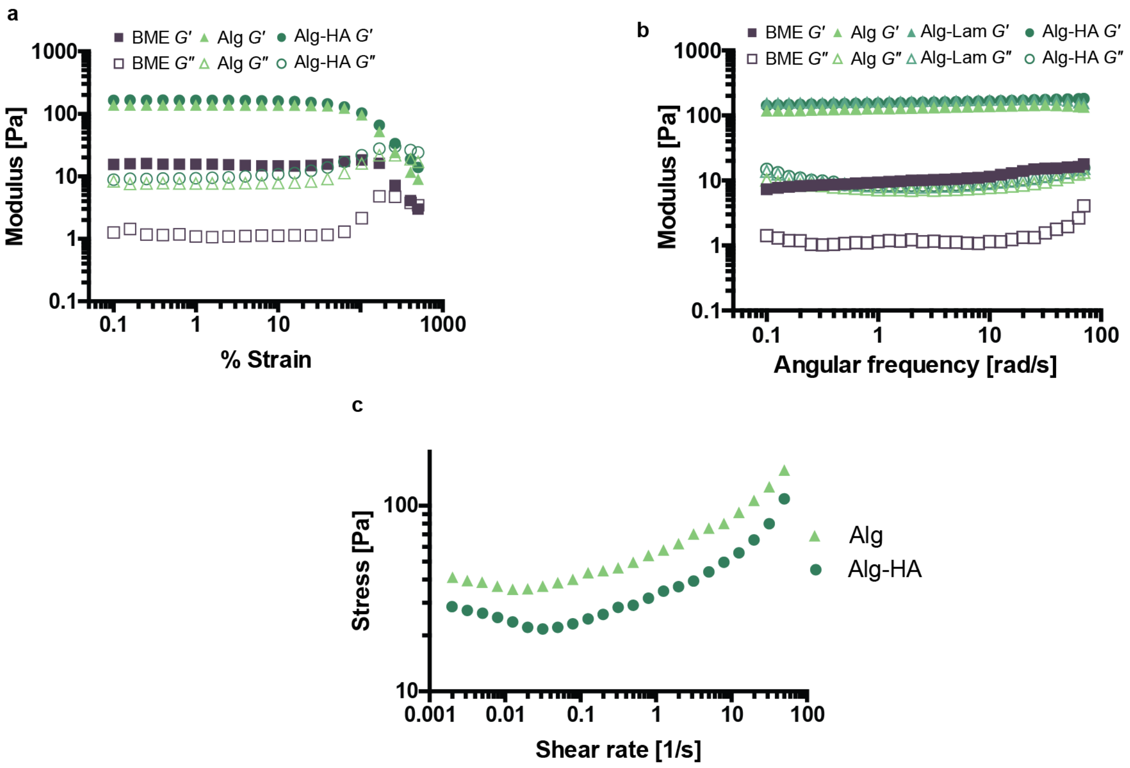

Supplementary Figure 1: a, Amplitude sweep (w=10 rad/s) for all formulations at 37°C. All hydrogel formulations show a similar stiffness, a similar solid-like response, and a similar range of linear viscoelasticity across the measured strains. BME is less stiff, but yields at a similar strain. **b,** Frequency sweep (strain=1%) for all formulations at 37°C. All hydrogel formulations show a similar stiffness, a similar solid-like response, and a similar range of linear viscoelasticity across the measured frequencies. BME is less stiff, but demonstrates a similar solid-like response. **c,** Flow sweep for Alginate and Alg-HA formulations at 25°C. Both formulations show a yield stress of approximately 20 Pa.

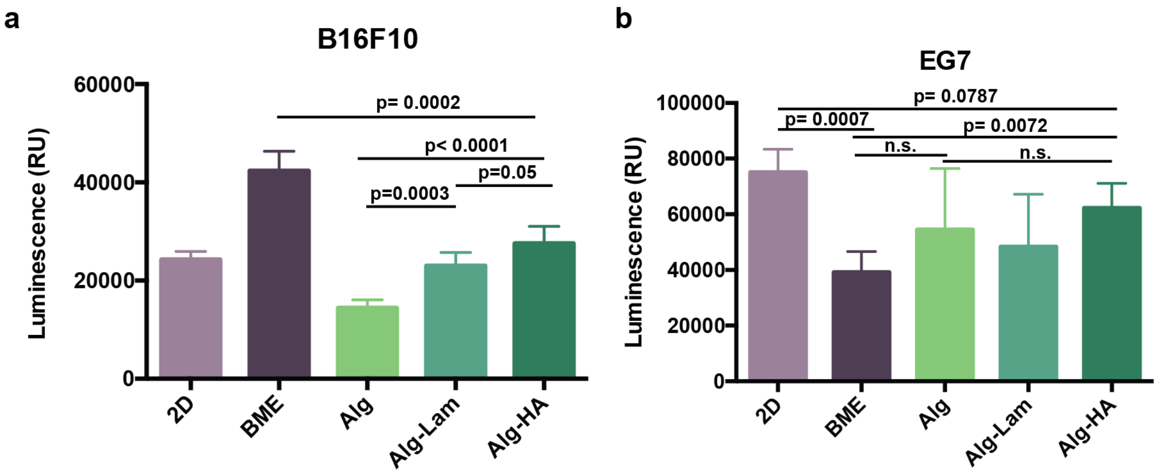

Supplementary Figure 2: a, Relative viability of B16F10 cells in various formulations after 1 day in culture after seeding 5,000 cells. Luminescent signal is linear to the cell number. **b,** Relative viability of of EG7 cells after seeding 10,000 cells after 1 day in culture in various formulations. Error is reported as non-significant (n.s.) if $p < 0.05$.

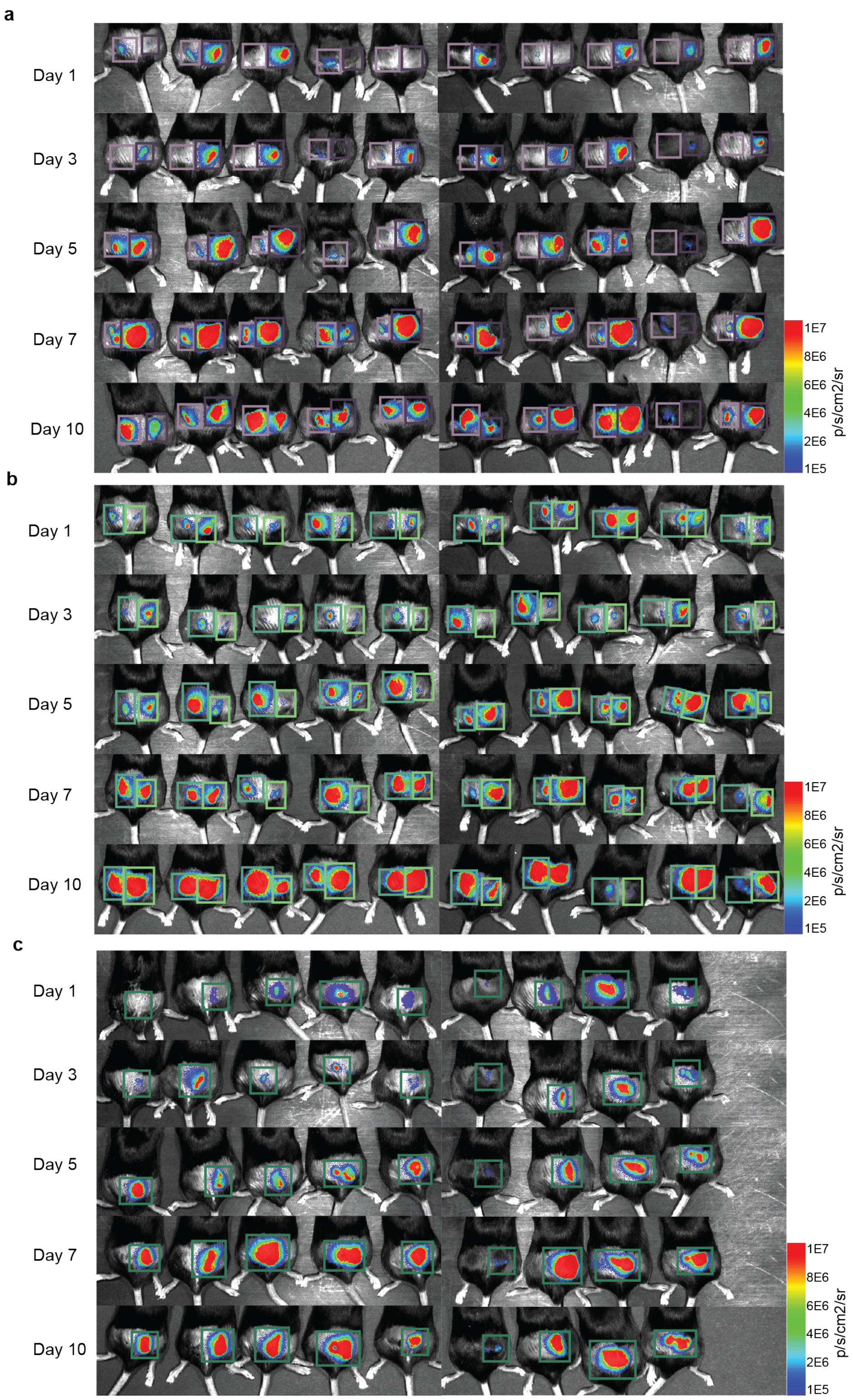

Supplementary Figure 3: *In vivo* imaging over time of mice with luminescent tumors forming. Tumors were innoculated with cells dispersed in saline, BME, Alg, Alg-Lam and Alg-HA. **a,** Saline (left, light purple) and BME (right, dark purple) groups. **b,** Alginate-Laminin (left, medium green) and Alginate (right, light green) groups. **c,** Alginate-Hyaluronic Acid (center, dark green) group.

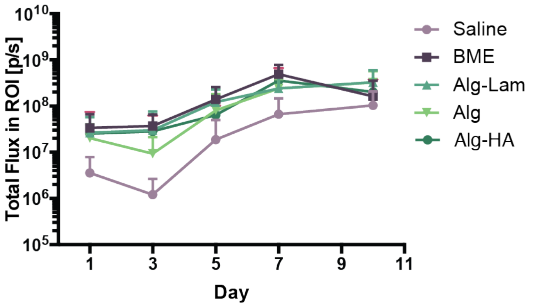

Supplementary Figure 4: The average total flux in region of interest surrounding the tumor of interest and standard deviation from in vivo imaging on various days directly after inoculation for all experimental groups.

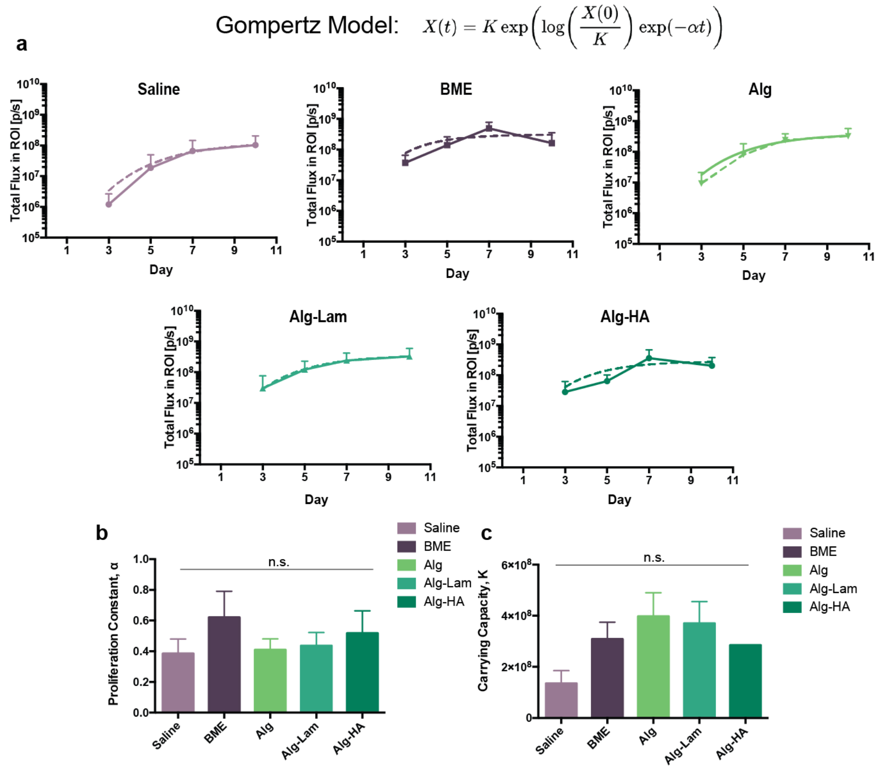

Supplementary Figure 5: *In vivo* imaging fit to Gompertz model. Parameter α that represents the proliferation constant, parameter K that represents the carrying capacity, *X(0)* represents the initial size, *t* represents time (days). **a,** Tumor luminescence fits to Gompertz equation after initial delivery phase (starting on Day 3, see methods for details). **b,** Parameter α in the Gompertz model and standard error calculated from the fit for all groups. **c,** Parameter K from the Gompertz model and standard error calculated from the fit for all groups.

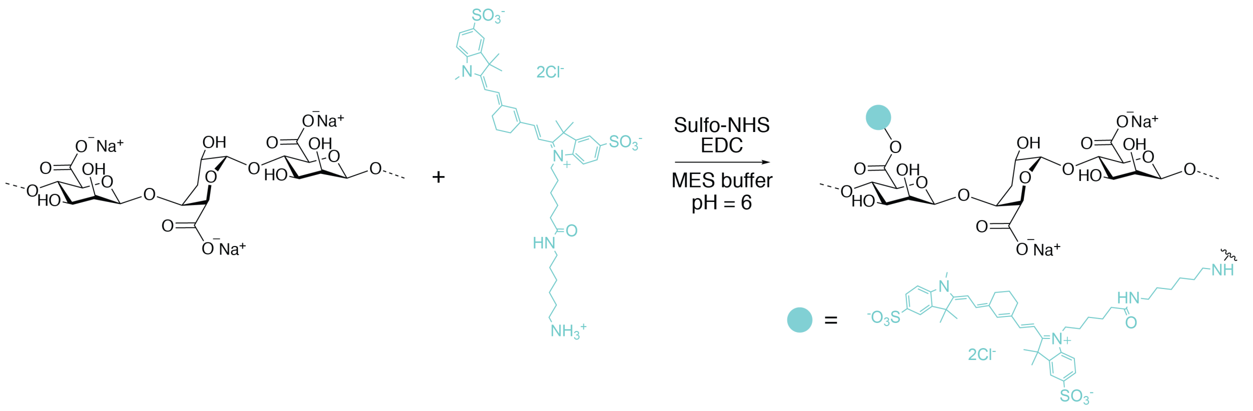

Supplementary Figure 6: Cy7 dye conjugation to alginate using carbodiimide chemistry.

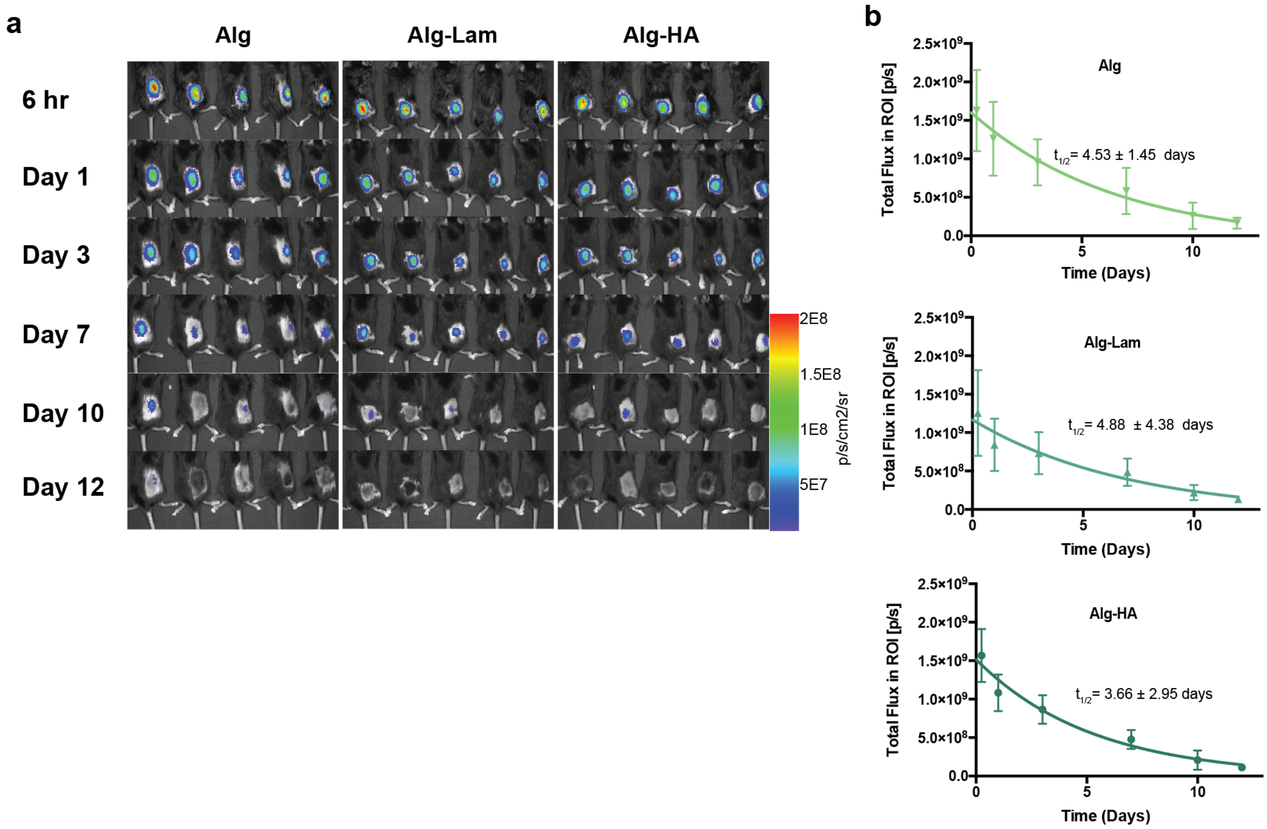

Supplementary Figure 7: a, *In vivo* degradation of fluorescent alginate measured using an *in vivo* imaging system for all hydrogel groups. B16F10 cells were encapsulated and co-injected in all groups as in previous studies. **b,** Average total flux and standard deviation from the region of interest surrounding the injection over time. A one-phase exponential decay was fitted to each curve and a half-life was computed for each group as shown on the graph.

Supplementary Table 1: P-values calculated with two-sided t-tests from analysis of tumor areas for all groups (Fig 2).

Group Pair	Day 10	Day 13	Day 15
Saline, BME	0.0002	0.0011	0.0045
Saline, Alg	<0.0001	0.0239	0.2778
Saline, Alg-Lam	<0.0001	0.0014	0.0290
Saline, Alg-HA	<0.0001	0.0015	0.0027
BME, Alg	0.5848	0.0163	0.0076
BME, Alg-Lam	0.9994	0.2423	0.0978
BME, Alg-HA	0.2800	0.2251	0.3943
Alg, Alg-Lam	1.0000	0.0282	0.0501
Alg, Alg-HA	1.0000	0.0113	0.0003
Alg-Lam, Alg-HA	0.1637	0.9485	0.1133

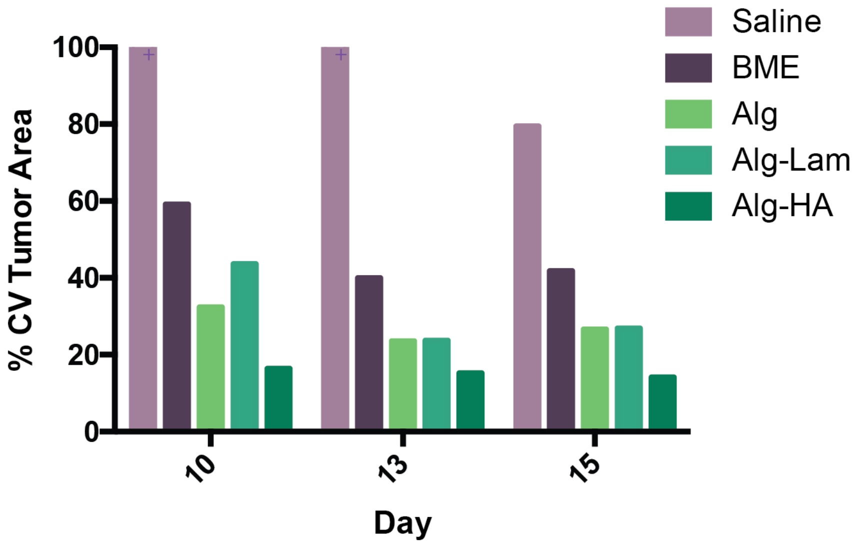

Supplementary Figure 8: %CV of tumor area for all groups: saline, BME, Alg, Alg-Lam and Alg-HA. Same data as Fig 2a plotted on one graph to show direct comparisons. Saline exceeded an 100 %CV on Days 10 and 13.

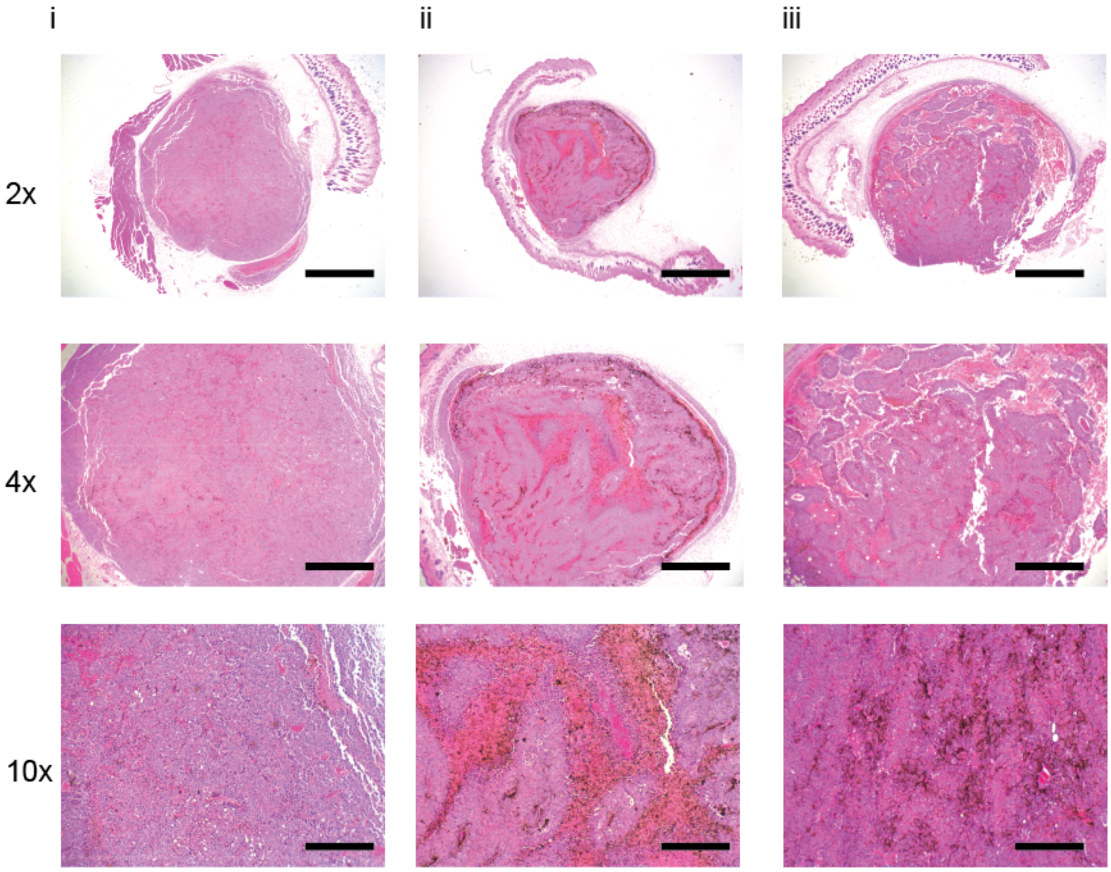

Supplementary Figure 9: Additional histology images with Hemotoxylin and Eosin staining from Day 15 for tumors formed in saline. Columns represent a single tumor, and rows represent a single magnification. Saline tumors were smaller than other groups. 2x magnification the scale bars represent 2 mm. 4x magnification the scale bars represent 1 mm. 10x magnification the scale bars represent 400 um.

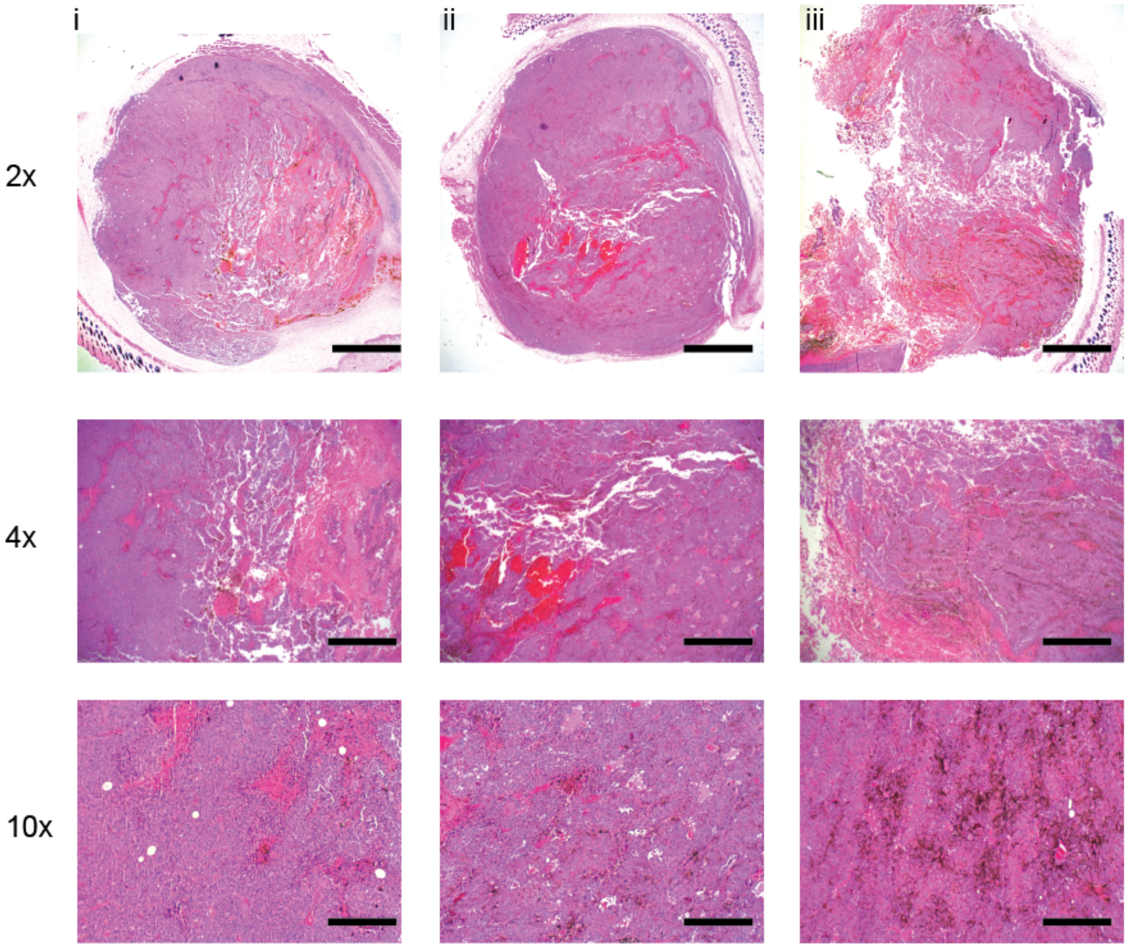

Supplementary Figure 10: Additional histology images with Hemotoxylin and Eosin staining from Day 15 for tumors formed in BME. Columns represent a single tumor, and rows represent a single magnification. BME formed the largest tumors of all groups. 2x magnification the scale bars represent 2 mm. 4x magnification the scale bars represent 1 mm. 10x magnification the scale bars represent 400 um.

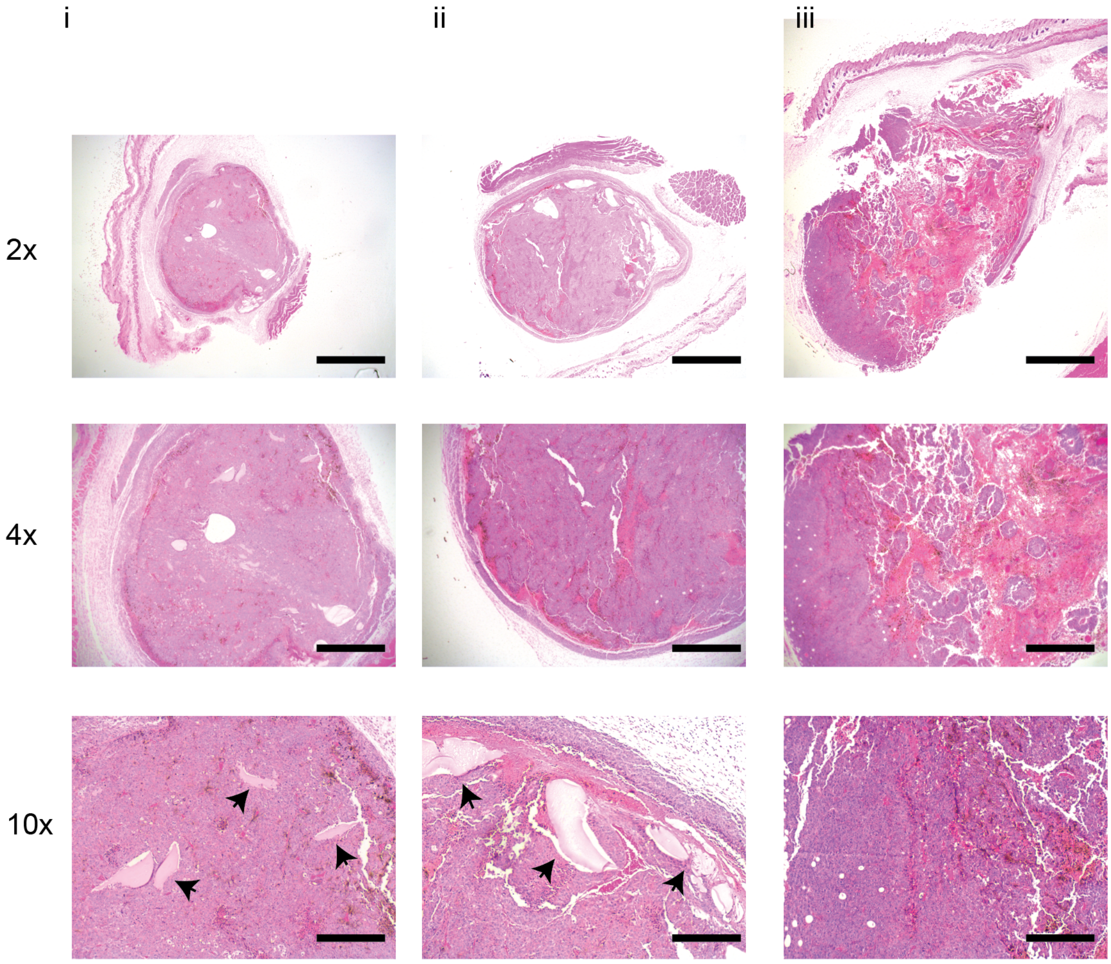

Supplementary Figure 11: Additional histology images with Hemotoxylin and Eosin staining from Day 15 for tumors formed in Alginate. Microscopic examination by a pathologist confirmed that the melanomas demonstrated identical histomorphologic features across all formulations. There is amorphous, pale eosinophilic material additionally present in some tumors formed in gels that may represent residual gel material. This material is designated by black arrows. Upon evaluation by a blinded pathologist, these tumors do not appear morphologically different than tumors formed in BME and saline. These tumors formed in alginate were smaller than the other gel groups. Columns represent a single tumor, and rows represent a single magnification. 2x magnification the scale bars represent 2 mm. 4x magnification the scale bars represent 1 mm. 10x magnification the scale bars represent 400 um.

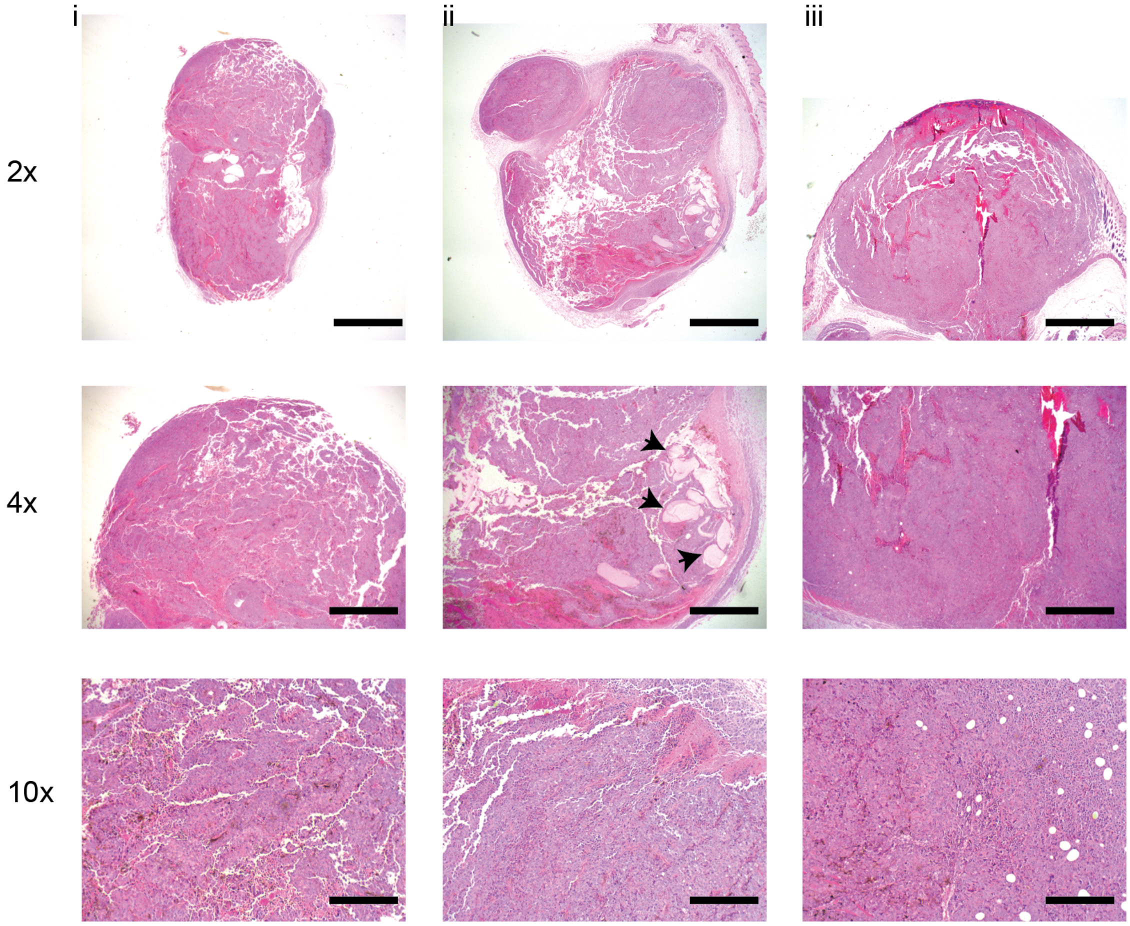

Supplementary Figure 12: Additional histology images with Hemotoxylin and Eosin staining from Day 15 for tumors formed in Alginate-Laminin. There is amorphous, pale eosinophilic material additionally present in some tumors formed in gels that may represent residual gel material. This material is designated by black arrows. Upon evaluation by a blinded pathologist, these tumors do not appear morphologically different than tumors formed in BME and saline. Columns represent a single tumor, and rows represent a single magnification. 2x magnification the scale bars represent 2 mm. 4x magnification the scale bars represent 1 mm. 10x magnification the scale bars represent 400 um.

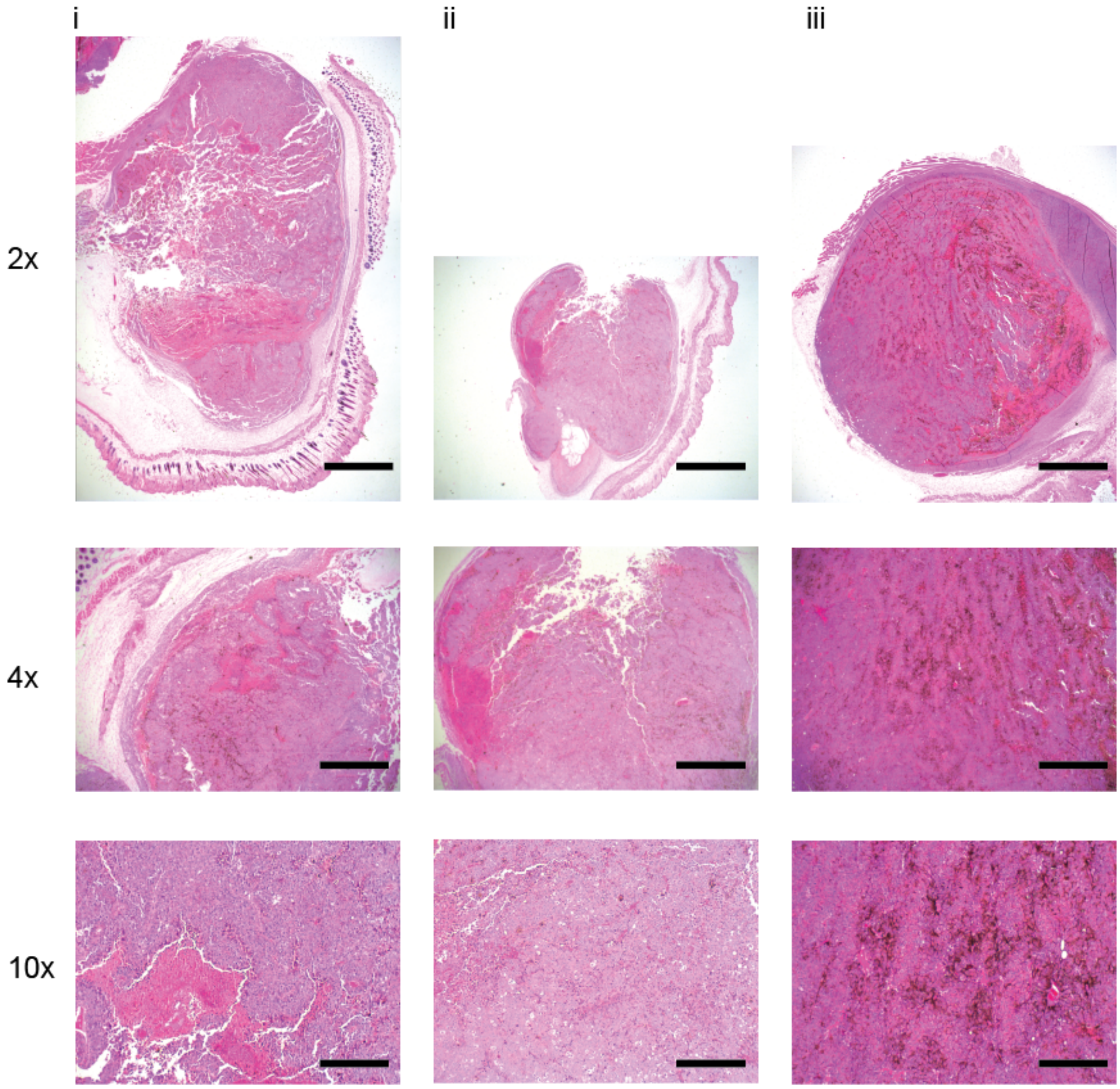

Supplementary Figure 13: Additional histology images with Hemotoxylin and Eosin staining from Day 15 for tumors formed in Alginate-Hyaluronic Acid. Upon evaluation by a blinded pathologist, these tumors do not appear morphologically different than tumors formed in BME and saline. Tumors formed in Alg-HA were the largest on averge of all the gel groups. Columns represent a single tumor, and rows represent a single magnification. 2x magnification the scale bars represent 2 mm. 4x magnification the scale bars represent 1 mm. 10x magnification the scale bars represent 400 um.

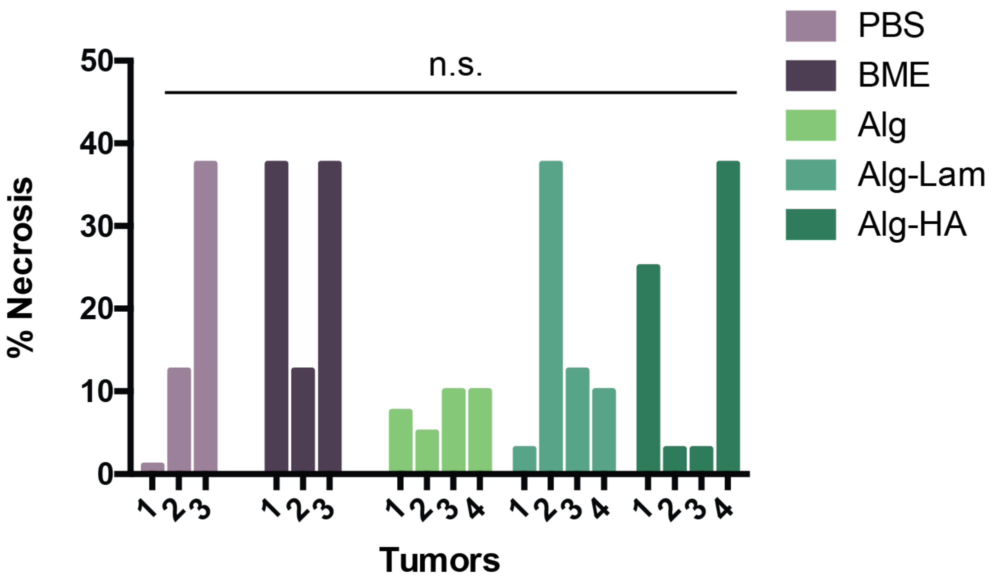

Supplementary Figure 14: % Necrosis as determined by a blinded pathologist for each tumor in all experimental groups.

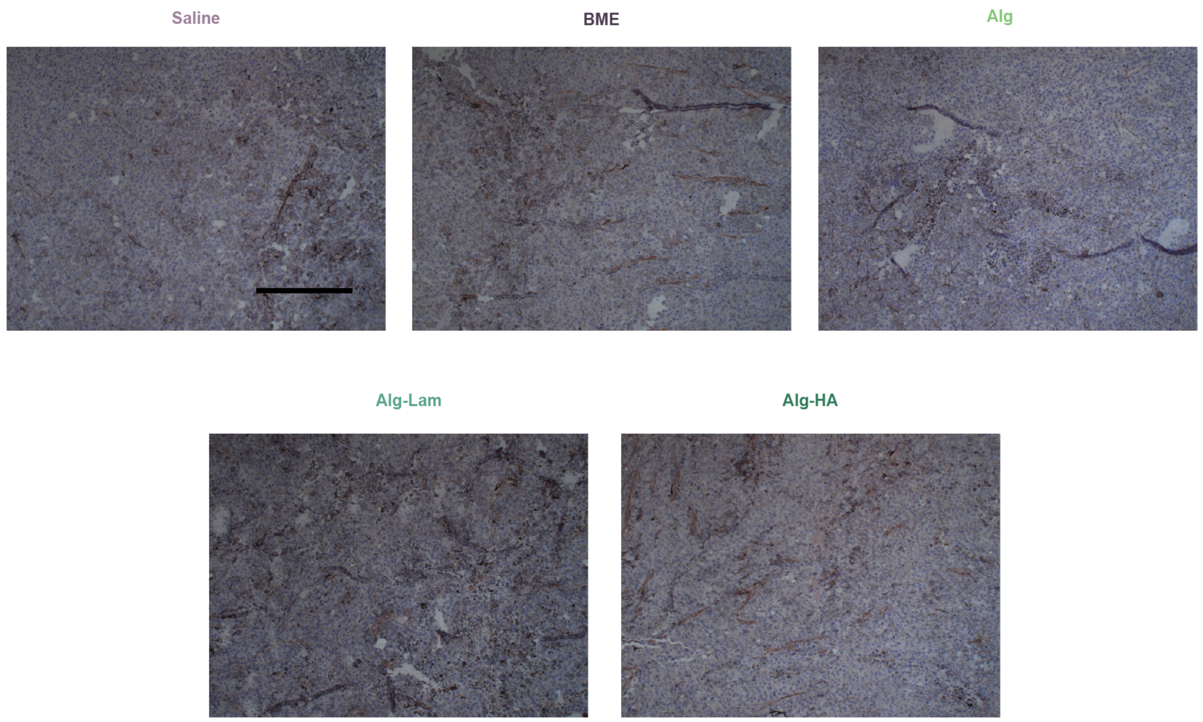

Supplementary Figure 15: Representative CD31 staining from each group. No significant differences were observed by a blinded pathologist. Background melanin made clear analysis challenging. Scale bar represents 250 um.

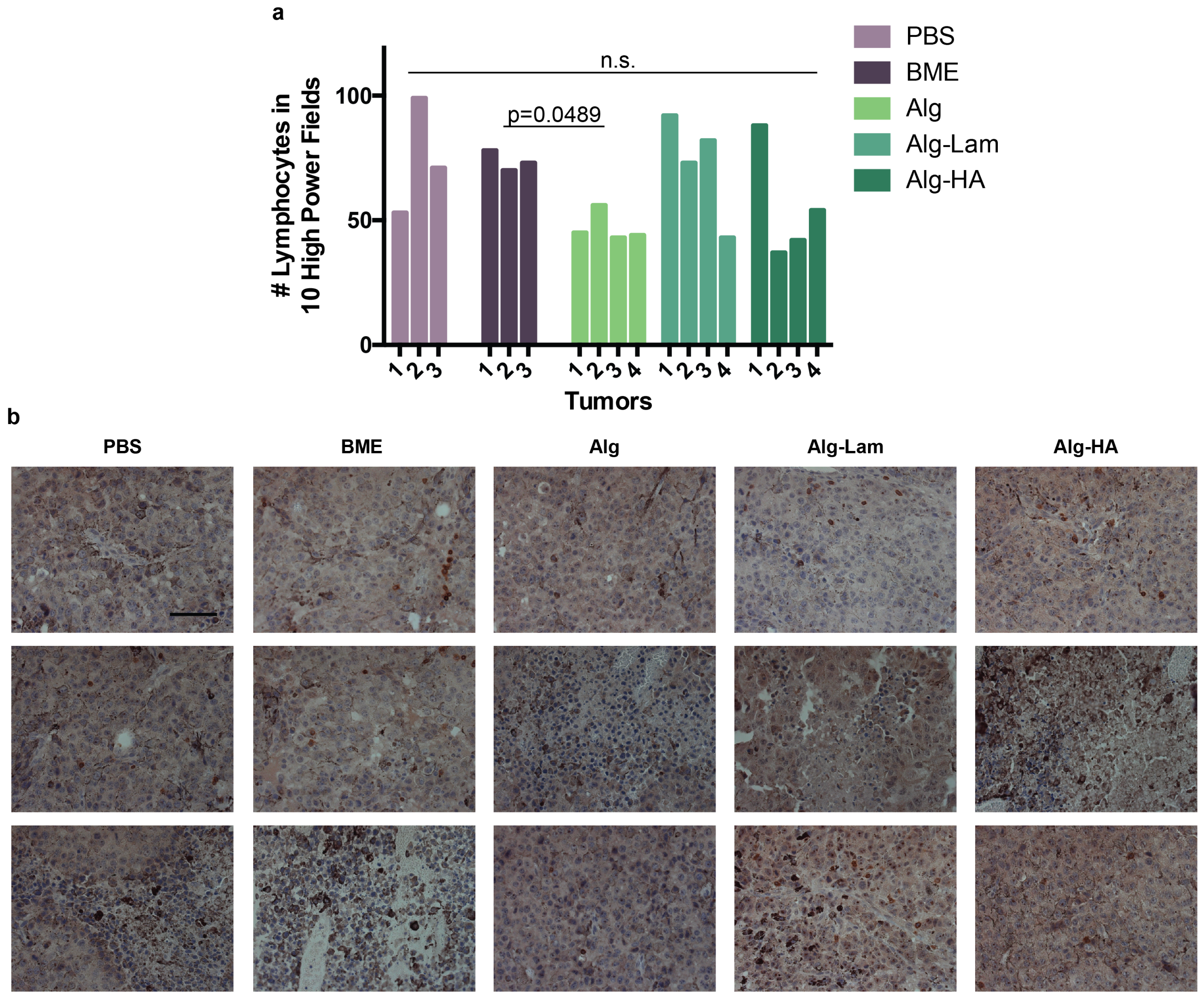

Supplementary Figure 16: **a,** Number of lymphocytes per tumor found in 10 high power fields (40x magnification) for all experimental groups. **b,** Representative high power images of CD3 stained tumors where lymphocytes are stained dark brown. Scale bar represents 50 um.

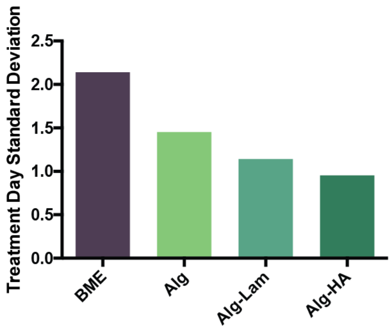

Supplementary Figure 17: Standard deviation of treatment day for each formulation. Standard deviations from the averages shown in Fig 3c.

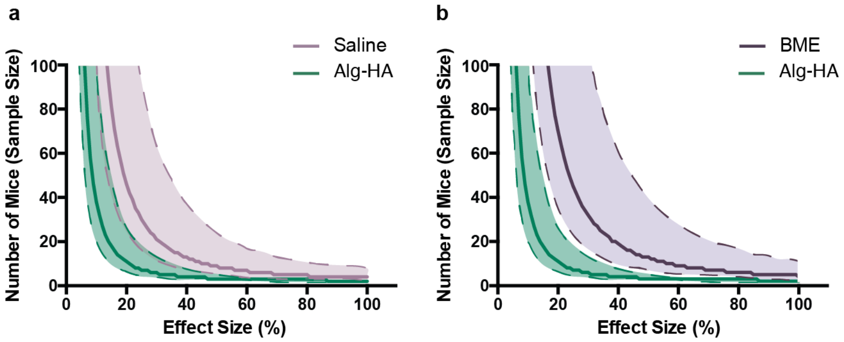

Supplementary Figure 18: a-b, Power analysis predicting the number of mice per group needed based off the experimental data obtained with 80% power. The dashed lines and shading represent the high and low number estimate of mice given the high and low 95% confidence interval values on the input coefficient of variance for these power calculations. Confidence intervals on coefficient of variation were calculated using Vangel's modification on McKay's theory on coefficient of variance. The Statistical toolbox in Matlab was used for power calculations, specifically the *sampsizepwr* function with two-sided t tests. The average and distribution of the day that each formulation surpassed 100 mm^2 were used in the power analysis.

Sample Matlab calculations for power analysis calculations

```
%Fig 3d
Power2 = 0.8;
End = 100;
samplesizePBS2 = [];
for x = 0:0.01:End
n = sampsizepwr('t2',[averagePBS_day15 , stdPBS_day15], x*averagePBS_day15, ...
    Power2,[]);
samplesizePBS2 =  [ samplesizePBS2 n];
end
samplesizebme2 = [];
for x = 0:0.01:(End)
n = sampsizepwr('t2',[averagebme_day13 , stdbme_day13], x*averagebme_day13, ...
    Power2,[]);
samplesizebme2 =  [samplesizebme2 n];
end
samplesizehighHA2 = [];
for x = 0:0.01:End
n = sampsizepwr('t2',[averagehighHA_day13 , stdhighHA_day13], ...
    x*averagehighHA_day13, Power2,[]);
samplesizehighHA2 =  [ samplesizehighHA2 n];
end

%Fig 3e
End = 50;
samplesize1 = [];
for x = 1:1:End
n = sampsizepwr('t2',[100, x], 60, Power2,[]);
samplesize1 =  [samplesize1 n];
end
samplesize2 = [];
for x = 1:1:End
n = sampsizepwr('t2',[100, x], 70, Power2,[]);
samplesize2 =  [samplesize2 n];
end
samplesize3 = [];
for x = 1:1:End
n = sampsizepwr('t2',[100, x], 80, Power2,[]);
samplesize3 =  [samplesize3 n];

%Fig 3f
power1 = [];
for x = 1:1:End
pwrout = sampsizepwr('t2',[100, x], 80,[],10);
pwrout = pwrout*100;
power1 =  [power1 pwrout];
end
power2 = [];
for x = 1:1:End
pwrout = sampsizepwr('t2',[100, x], 70,[],10);
pwrout = pwrout*100;
power2 =  [power2 pwrout];
end
power3 = [];
for x = 1:1:End
pwrout = sampsizepwr('t2',[100, x], 60,[],10);
pwrout = pwrout*100;
power3 =  [power3 pwrout];
end
```
