# Supplementary material for: Consistent tumorigenesis with self-assembled hydrogels enables high-powered murine cancer studies
Source: Commun Biol. 2021 Aug 19;4:985. doi: 10.1038/s42003-021-02500-8 (PMC8376877; doi:10.1038/s42003-021-02500-8)
Supplement: Supplementary file 3 — Description of Supplementary Files [file 42003_2021_2500_MOESM3_ESM.pdf]

## Description of Additional Supplementary Files

**File name:** Supplementary Video 1

**Description:** *Formulation of cargo-loaded alginate hydrogels by simple mixing.* The hydrogel is prepared with a dual syringe mixer whereby a 5 wt.% polymer solution is loaded in one syringe (right), and cells and 10 mM calcium sulfate are placed in the second syringe (left, dyed blue for this demonstration for easier viewing). The two solutions are mixed with a female-female dual syringe mixer by pumping the material back and forth for 30 seconds to form a robust hydrogel that is already loaded into a syringe and ready for administration.

**File name:** Supplementary Movie 2

**Description:** *Injectability of cargo-loaded self-assembled hydrogels.* These alginate-based hydrogels are easily injected from standard syringes after mixing and loading into syringes. The mechanical properties of the hydrogel, which is dyed blue for this demonstration for easier viewing, prevent settling of cargo and local retention in a depot following injection.

Caption: Data from main text manuscript figures is available at https://doi.org/10.6084/m9.figshare.14813880.v1.
