# Supplementary material for: Consistent tumorigenesis with self-assembled hydrogels enables high-powered murine cancer studies
Source: Commun Biol. 2021 Aug 19;4:985. doi: 10.1038/s42003-021-02500-8 (PMC8376877; doi:10.1038/s42003-021-02500-8)
Supplement: Supplementary file 6 — Reporting Summary [file 42003_2021_2500_MOESM6_ESM.pdf]

Corresponding author(s): Eric Appel

Last updated by author(s): Jul 12, 2021

Reporting Summary

Nature Research wishes to improve the reproducibility of the work that we publish. This form provides structure for consistency and transparency in reporting. For further information on Nature Research policies, see our Editorial Policies and the Editorial Policy Checklist.

Statistics

For all statistical analyses, confirm that the following items are present in the figure legend, table legend, main text, or Methods section.

n/a	Confirmed	
☐	☒	The exact sample size (*n*) for each experimental group/condition, given as a discrete number and unit of measurement
☐	☒	A statement on whether measurements were taken from distinct samples or whether the same sample was measured repeatedly
☐	☒	The statistical test(s) used AND whether they are one- or two-sided<br>*Only common tests should be described solely by name; describe more complex techniques in the Methods section.*
☒	☐	A description of all covariates tested
☒	☐	A description of any assumptions or corrections, such as tests of normality and adjustment for multiple comparisons
☐	☒	A full description of the statistical parameters including central tendency (e.g. means) or other basic estimates (e.g. regression coefficient) AND variation (e.g. standard deviation) or associated estimates of uncertainty (e.g. confidence intervals)
☒	☐	For null hypothesis testing, the test statistic (e.g. *F*, *t*, *r*) with confidence intervals, effect sizes, degrees of freedom and *P* value noted<br>*Give P values as exact values whenever suitable.*
☒	☐	For Bayesian analysis, information on the choice of priors and Markov chain Monte Carlo settings
☒	☐	For hierarchical and complex designs, identification of the appropriate level for tests and full reporting of outcomes
☐	☒	Estimates of effect sizes (e.g. Cohen's *d*, Pearson's *r*), indicating how they were calculated

Our web collection on statistics for biologists contains articles on many of the points above.

Software and code

Policy information about availability of computer code

Data collection	For most studies, software was not used to acquire data. Aura imaging software was used to acquire bioluminescence data.
Data analysis	GraphPad Prism 6 (GraphPad Software) was used for statistical analysis. Matlab (Statistical Toolbox) was used for statistical analysis and power calculations.

For manuscripts utilizing custom algorithms or software that are central to the research but not yet described in published literature, software must be made available to editors and reviewers. We strongly encourage code deposition in a community repository (e.g. GitHub). See the Nature Research guidelines for submitting code & software for further information.

Data

Policy information about availability of data

All manuscripts must include a data availability statement. This statement should provide the following information, where applicable:

- Accession codes, unique identifiers, or web links for publicly available datasets
- A list of figures that have associated raw data
- A description of any restrictions on data availability

All data supporting the results in this study are available within the Article and its Supplementary Information. The broad range of raw datasets acquired and analysed (or any subsets of it), which for reuse would require contextual metadata, are available from the corresponding author on reasonable request.

Field-specific reporting

Please select the one below that is the best fit for your research. If you are not sure, read the appropriate sections before making your selection.

- [x] Life sciences
- [ ] Behavioural & social sciences
- [ ] Ecological, evolutionary & environmental sciences

For a reference copy of the document with all sections, see nature.com/documents/nr-reporting-summary-flat.pdf

Life sciences study design

All studies must disclose on these points even when the disclosure is negative.

Sample size	n=10 or n=9 was chosen as the sample size for in vivo studies based on precedent of in vivo studies involving preclinical tumor models.
Data exclusions	No data was excluded.
Replication	Adequate sample sizes were used to assess reproducibility for in vivo results. For mechanical testing, multiple gels were formulated on different days to ensure reproducibility.
Randomization	Cages of mice were selected arbitrarily to receive different formulations.
Blinding	Data collection and analysis was performed blinded for all in vivo experiments. Histopathology data analysis was performed by a blinded professional pathologist without knowledge of samples.

Reporting for specific materials, systems and methods

We require information from authors about some types of materials, experimental systems and methods used in many studies. Here, indicate whether each material, system or method listed is relevant to your study. If you are not sure if a list item applies to your research, read the appropriate section before selecting a response.

Materials & experimental systems

n/a	Involved in the study	
x		Antibodies
	x	Eukaryotic cell lines
x		Palaeontology and archaeology
	x	Animals and other organisms
x		Human research participants
x		Clinical data
x		Dual use research of concern

Methods

n/a	Involved in the study	
x		ChIP-seq
x		Flow cytometry
x		MRI-based neuroimaging

Eukaryotic cell lines

Policy information about cell lines

Cell line source(s)	Luc+ B16F10 cells were purchased from ATCC (ATCC CRL-6475-LUC2).
Authentication	Cell lines were not authenticated.
Mycoplasma contamination	Cells tested negative for mycoplasma using Lonza Mycolert Mycoplasma Detection Kit.
Commonly misidentified lines (See ICLAC register)	N/A

Animals and other organisms

Policy information about studies involving animals; ARRIVE guidelines recommended for reporting animal research

Laboratory animals	Female 6-10 week old C57BL/6 mice from Charles River Laboratory were used for all experiments.
Wild animals	N/A
Field-collected samples	N/A
Ethics oversight	The animal studies were performed in accordance with the guidelines for the care and use of laboratory animals, and all protocols were approved by the Stanford Institutional Animal Care and Use Committee.

Note that full information on the approval of the study protocol must also be provided in the manuscript.
